# Perceived stress and social support as correlates of sickle cell anaemia severity in a low-resource setting

**DOI:** 10.1186/s12887-025-05572-1

**Published:** 2025-03-19

**Authors:** Ngozi Nancy Onu, Isaac Nwabueze Asinobi, Ikenna Kingsley Ndu, Anthony Nnaemeka Ikefuna, Tobechukwu Chinenye Ezike, Justus Uchenna Onu

**Affiliations:** 1 Department of Paediatrics, Enugu State University Teaching Hospital, Enugu, Nigeria; 2 Department of Paediatrics, Enugu State University of Science and Technology, Enugu, Nigeria; 3 Department of Paediatrics, University of Nigeria, Enugu Campus, Enugu, Nigeria; 4 Department of Mental Health, Nnamdi Azikiwe University, Nnewi Campus, Azikiwe, Nigeria

## Abstract

**Background:**

The role of biological factors such as foetal haemoglobin in modifying the severity of Sickle Cell Anaemia (SCA) has been extensively investigated. However, the phenotypic variability of SCA cannot be explained by genetic factors alone. Hence, the need to determine other correlates of SCA severity such as perceived stress and social support.

**Methods:**

This was a hospital-based cross-sectional study involving 60 participants aged 8–17 years selected using simple random sampling technique. Standard rating scales (i.e., SCA scoring system, perceived stress scale, and child and adolescent social support scale) were used to assess the severity of SCA, perceived stress and social support, respectively. Foetal haemoglobin (HbF) was estimated using high performance liquid chromatography. The psychosocial predictors of sickle cell severity were analyzed using multivariate linear regression.

**Results:**

There was a negative moderate significant correlation between SCA severity and domains of social support: parents’ (rho = -0.30, *p* = 0.03) and classmates’ (rho = -0.50, *p* < 0.001). Of all the variables studied, only classmates’ perceived social support emerged as the significant predictor of SCA severity when other confounders such as HbF were controlled for (β = -0.37, t = 2.41, *p* = 0.02).

**Conclusion:**

The findings of this study support the available literature on the mediating effect of social support on SCA severity. These findings buttress the need for clinicians to consider psychosocial methods (e.g., family and classmates’ support) in improving disease outcomes.

**Supplementary Information:**

The online version contains supplementary material available at 10.1186/s12887-025-05572-1.

## Introduction

Sickle Cell Anaemia (SCA) has significant public health implications due to its high morbidity and mortality [1]. In addition, it poses great psychological stress and financial burden on the caregivers of affected children [2, 3]. Worldwide, more than 300,000 children are born with SCA annually [4, 5]. Sub-Saharan Africa contributes over 75% of the global burden with an under five mortality rate up to 16% in some countries [5]. In Nigeria, about 150,000 babies are born with the disease annually, with a prevalence of about 2% [5].

A significant challenge to the effective management of patients with SCA is its substantial clinical heterogeneity ranging from near complete asymptomatic illness to severe debilitating illness [6–9]. This clinical heterogeneity has been linked to several factors broadly classified into genetic and environmental factors [7–9]. Foetal haemoglobin (HbF) is one of the most extensively studied genetic correlates of sickle cell severity [8]. Environmental factors including psychosocial variables have, however, also been implicated as determinants of severity among children with SCA [8–11].

With the advent of the biopsychosocial model as popularized by Engel, emphasis is now on psychological and social mediators of health and disease [12]. This biopsychosocial approach supports the integration of biological, psychological and social factors in the assessment, prevention and treatment of diseases and thus offers a more holistic approach to a patient [13, 14]. This is in contrast with the biomedical model which explains illness in terms of single-factor biological malfunction with little attention to behavioral and social processes [14]. Psychological stress and psychosocial support are known to modulate chronic illnesses generally, and SCA specifically [13, 15, 16]. Some studies have found a significant association between perceived stress and indices of disease severity such as increased number of vaso-occlusive crises, reduced activity, increased utilization of healthcare services and school absenteeism [17–21].

Better quality of life, psychological well-being, and a milder severity of SCA have been linked to satisfactory perceived social support [15, 16, 22]. There is a distinction between available social resources (structural) and the individuals’ subjective perception or assessment of received support (functional) [23]. Both forms of social support have been reported to be protective against distress and adverse outcome [23]. More so, perceived and tangible social supports have been reported to mediate disease severity and improve quality of life [15, 16, 22].

The strict biomedical perspective of diseases has been viewed by critics as narrow and reductionist in approach [14]. The importance of psychological and socio-cultural factors is now well recognized in the general medical practice. Globally, there is paucity of data on the psychological and socio-cultural factors that influence SCA despite the extensive research on the effect of biological and other clinical variables [9, 24–27]. In the light of the above issues, the main objective of this study was: to determine the relationship between psychosocial variables (e.g., perceived stress and perceived social support) and SCA severity.

## Materials and methods

### Study design and setting

This was a cross sectional study carried out at the Paediatric Sickle Cell Clinic of a Nigerian tertiary institution. The Sickle Cell clinic has a population of about 300 children, of which about 160 are aged 8 to 17 years. The hospital has laboratory services for haematology, microbiology, and clinical chemistry investigations.

### Ethical considerations

#### Ethical approval

was obtained from the Health Research and Ethics Committee of the institution, with reference number UNTH/NREC/2021/06/226. International ethical norms and standards were strictly adhered to; written informed consent was obtained from parents/guardians of the participants. Assent was obtained from all the participants. The participants were also informed that if in the process of the research, any previously undiagnosed clinical condition was found, the managing team would be appropriately informed. Information gathered in the course of the research was stored in an encrypted computer accessible only to the researcher.

### Sample size estimation

With a trait prevalence of 25%, or one in every four persons, an estimated 4-6 million people in Nigeria are believed to have sickle cell disease [28]. However, in the study setting, about 160 children with SCA aged eight to 17 years were seen annually. In computing the required sample size for the study, we used the findings of Diwe et al. [29] on the prevalence of SCA in the South Eastern region of Nigeria of 5%. Based on their finding, we computed the required sample size by using these figures to substitute in the Cochran formula for a single proportion ($\:n=\frac{{z}^{2}\left(p\right)\left(q\right)}{{e}^{2}}$) [30] and arrived at 60, which was judged to have sufficient power to answer the research question.

### Sample selection

The list of SCA patients aged 8-17years registered with the Sickle Cell Clinic of the institution was obtained from the records department. A computer-generated table of random numbers was used to select prospective participants (i.e., simple random sampling technique). The randomly selected patients were communicated prior to their scheduled routine follow up date. Those who missed this date were followed up and a subsequent visit was scheduled. Replacements were made if the selected person did not meet the study criteria or refuses to consent or give assent to participate.

Participants were included if they were diagnosed with SCA using alkaline-acid haemoglobin electrophoresis and aged eight (8) to seventeen (17). Those with chronic co-morbidities such as asthma, diabetes, malignancy, renal disease, heart disease and intellectual disabilities such as autism, cerebral palsy, hearing loss, speech problems that may interfere with the patient’s understanding and answering of interview questions were excluded.

### Data collection procedure

#### Obtaining consent/assent

Recruitment was done as the randomly selected patients present to the sickle cell clinic of the study setting. Details of the study protocol were explained to prospective participant/caregiver pair in clear terms when they presented to the SCA clinic. The voluntariness of participation was emphasized, and patients/caregivers were assured that refusal to participate will not affect their medical care. Caregivers who were willing to participate gave consent either in writing or by thumb printing. Verbal assent was obtained from children of the consenting caregivers.

### Questionnaire administration

After consent was obtained, a thorough history, physical and mental state examination was done to exclude ineligible participants. Thereafter, those who met the study eligibility criteria were recruited. The researcher obtained socio-demographic and clinical data using the proforma and in some cases the medical records. Each participant was given the perceived stress and perceived social support scales to assess their stress level and social support, respectively. Severity of SCA was assessed using the SCA scoring system. Participants’ socio-economic class was categorized according to the criteria described by Oyedeji [31].

#### The perceived stress scale (PSS)

The PSS is one of the most widely used psychological instrument for measuring the perception of stress and is a robust predictor of health and disease [32]. It is a self-reported, 10-item questionnaire scored using a 5-point Likert scale. Scores range from 0 to 40 with higher scores indicative of higher stress [32, 33]. The PSS has strong psychometric properties, possessing good reliability and construct validity among multicultural populations [34, 35]. Although the PSS is designed for use in subjects with at least a junior high school education, the National Institutes of Health Toolbox for the Assessment of Neurological and Behavioral Function initiative recommends using the 10-item version for children aged 12–17 years (self-report) and parents of children aged 8–12 years (proxy-report) [36]. The scale has been used in adolescent sickle cell patients and has also been used in Nigerian populations [37–40].

#### The child and adolescent social support scale (CASSS)

The CASSS is a well-validated and reliable tool used to assess the perceived social support of children and adolescents [41]. The CASSS is a 60-item multidimensional scale measuring perceived social support from five sources: parents, teachers, classmates, close friends and people in the school [41]. Each item is a statement of the four types of support (emotional, informational, appraisal and instrumental). The revised version (CASSS 2000) has only one version suitable for children aged 8 to 18years. The children are asked to rate how often they perceive the support and how important it is to them that they perceive the support. Frequency ratings are on a 6-point Likert scale (ranging from 1 = Never to 6 = Always). Importance ratings are on a 3-point scale (ranging from 1 = not important to 3 = very important). Subscale scores are calculated by summing the frequency ratings of the 12 items on each subscale (Parent, Teacher, Classmate, Close Friend and School). Higher scores indicate higher perception of support. The CASSS has been well studied with strong psychometric properties and has been used in children with sickle cell anaemia [7, 22].

### Assessment of sickle cell anaemia severity

A comprehensive scoring system of SCA severity based on clinic-laboratory variables was developed by Adegoke and Kuti [9]. It assesses a total of 15 parameters to reflect each patient’s present state, their state during the previous 12 months, and lifetime complications [9]. Items are scored according to the frequency of occurrence and severity, with scores ranging from 1 to 5. Acute life-threatening events and neurological complications are assigned higher scores; for instance, cerebrovascular disease (CVD) is assigned a score of 5, and pneumococcal meningitis and acute chest syndrome (ACS) are assigned scores of 3. Avascular necrosis (AVN) is assigned a lower score because it does not immediately lead to acute life-threatening events. The total score is calculated for each child (range from 0 to 34), with higher scores indicating more severe disease. The scoring system was chosen for the following reasons: first, it is simple and has been robustly used in our environment. Secondly, factors from other chronic illnesses including kidney, cardiac, and lung disease were excluded from this scoring system, which if present in the index study could confound the experience of perceived stress.

### Blood collection and analysis

Venous blood samples were collected from all participants. This was used for HbF estimation and complete blood count (haemoglobin concentration/packed cell volume and white blood cell count). The materials (disposable gloves, tourniquet, sample bottles, sterile needles and syringes, plaster, 70% alcohol cotton swabs and dry cotton wool balls) required for the procedure were assembled in a tray. The test to be performed was discussed with participant/caregiver pair and verbal consent was obtained. The participants’ full names and hospital numbers were written on the investigation forms and Ethylenediaminetetraacetic acid (EDTA) bottles. The participants were placed comfortably in a sitting position and reassured. The venipuncture site was identified, and a clean tourniquet was applied 4–5 finger widths above this site. The investigator observed basic aseptic procedures. Appropriately sized needle was used with a syringe to collect five (5) milliliters of blood. The tourniquet was untied and firm pressure applied at venipuncture site with the dry cotton wool balls and the used needles were discarded into a sharp container. Three milliliters of blood were transferred to the first EDTA bottle for HbF assay while the remaining two milliliters were transferred to the second EDTA bottle for full blood count. The sample bottles were gently rocked to ensure mixing with the EDTA. The patient’s information on the bottles and the forms were cross-checked and the blood stored vertically at room temperature on a specimen rack dedicated for the study at the side laboratory till the end of the clinic day (maximum of eight hours). Thereafter, all samples collected for the day were dispatched to the Haematology laboratory of the Teaching Hospital. Foetal haemoglobin was assayed with an automated high performance liquid chromatography machine at the same laboratory using the following steps [42]: **Sample Preparation (Step 1)**: The blood sample was properly mixed and a fraction (i.e., about 100 µl) was transferred to the sample cup. **Haemolysis (Step 2)**: A haemolyzing agent, 0.1% saponin was added to the sample to facilitate the release of haemoglobin from the red cells. **Sample Injection (Step 3)**: The haemoyzed samples were loaded into the high-performance liquid chromatography (HPLC) machine’s autosampler. **Chromatographic Separation (Step 4)**: The HPLC machine separates the different haemoglobin fractions based on their ionic interactions with the stationary phase. **Detection and Quantitation (Step 5)**: The separated hemoglobin fractions are detected by their absorbance at 415 nm, and the HbF percentage was calculated based on the peak areas.

### Data analysis

Analysis of the results was done using the International Business Machine Statistical Package for the Social Sciences (IBM-SPSS, version 20, Armonk, NY: IBM Corp) [43]. Questionnaires were scrutinized for incorrectly filled information and then cleaned. The normality of distribution of data was done using Kolmogorov-Smirnov test. Spearman’s correlation was used to determine the direction, magnitude and significance of the relationship between the total severity scores and the total scores of perceived stress scale, perceived social support and subscale scores. Multivariate linear regression analysis was used to determine the psychosocial predictors of SCA severity while controlling for potential confounding variable such as age, social class, gender, and HbF. A test was considered significant if p was less than 0.05. All test were two-tailed at 95% confidence interval.

## Results

Participants were mostly early adolescents with mean age of 14 years, predominantly males (51.7%) with mild disease severity (Table 1). There was a negative moderate significant correlation between SCA severity scores and parents’ (rho_s_= -0.30, *p* = 0.03) and classmates’ social support (rho_s_= -0.50, *p* < 0.001) as shown in Table 2. Psychosocial variables as predictors of SCA severity is shown in Table 3. Classmates’ social support emerged as the predictor of SCA severity after controlling for socio-demographic variables of age, social class, sex and biological variable (i.e., HbF).

**Table 1:** Socio-demographic and clinical characteristics of the participants *N* = 60

Variables	Frequency (%)	Mean (SD)
Mean Age (years)		14.00 (2.48), Range: 9.00–17.00 years
Gender		
Male	31(51.7)	
Female	29(48.3)	
Total	**60(100.0)**	
Educational Status		
Primary	13 (21.7)	
Secondary	46 (76.7)	
Tertiary	1 (1.7)	
Total	**60(100.0)**	
Social Class		
Class 1	11(18.3)	
Class 2	15(25.0)	
Class 3	10(16.7)	
Class 4	24(40.0)	
Class 5	0(0.0)	
Total	**60(100.0)**	
SCA severity*		
Mild	35 (58.3)	
Moderate	25 (41.7)	
Severe	0 (0.0)	
Total	**60 (100.0)**	
Foetal Haemoglobin (%)		6.90 (5.50)**
Packed Cell Volume (%)		23.37 (4.13)
Complete White Blood Cell Count (cells/mm^3^)		11635.50 (4360.00)**
Platelet count (cells/mm^3^)		306000.00 (2100.50)**

NB: SD = Standard Deviation, *=severity scores (mild < 8, moderate 8–17, severe > 17)^9^, ** Median (interquartile range), Upper Class (Class I and II), Middle Class (Class III) and Lower Class (Class IV and V) [31]. Primary education in the index study (aged 8–11 years), secondary (aged 12–17 years), and tertiary (aged 17 years and above)

**Table 2:** Relationship between social support, perceived stress and severity of sickle cell anaemia

Variables	Sickle Cell Severity Score, Rho_s_ (*p*-value)
Social Support-Parents’ Sub-scale	-0.30 (0.03)
Social Support - Teachers’ Sub-scale	-0.23 (0.06)
Social Support - Classmates’ Sub-scale	-0.50 (< 0.001)
Social Support - Total Score	-0.40 (0.002)
PSS score	0.06 (0.67)

Rho = Spearman’s Correlation Coefficient, PSS = Perceived Social Support

**Table 3:** Multivariate linear regression analysis of the independent predictors of SCA severity

Dependent Variable	Independent predictors	Standard βcoefficient	t-test	*p*-value
Sickle Cell Severity Score	Foetal haemoglobin	-0.17	-1.15	0.26
	Classmates’ domain of social support	-0.37	-2.41	0.02
	Parents’ domain of social support	-0.04	-0.24	0.81
	Teachers’ domain of social support	-0.003	-0.02	0.99
	Perceived stress Score	-0.10	-0.69	0.50
	Age	-0.01	-0.11	0.91
	Gender	-0.11	-0.86	0.40
	Social class	-0.004	-0.03	0.98
	Platelet count	0.12	0.97	0.34

Coefficient of multiple determination (R^2^) = 0.125

## Discussion

The study aimed to determine the relationship between psychosocial variables and the severity of sickle cell disease among children and adolescents in a Nigerian Tertiary Hospital.

The main highlights of the findings of this study are: (1) Parental and classmates’ perceived social support was significantly associated with SCA severity; (2) perceived stress scores had a weak relationship with SCA severity; and (3) that classmates’ perceived social support emerged as the significant predictor of SCA severity after adjusting for age, social class and HbF.

The finding of the positive effect of parental and classmates’ perceived social support on the SCA severity is consistent with previous reports with other chronic diseases [15, 22, 44, 45]. The buffering role of social support in patients with chronic diseases such as SCA has been demonstrated in several studies [15, 22, 46, 47]. Researchers have also reported that children and adults with higher levels of social support have better health-related quality of life and less disease severity [15, 22, 46]. Classmates’ social support was the only significant predictor of disease severity obtained in the index study when other confounding variables were controlled for. This finding suggests that peer support is moderately correlated to disease severity. This beneficial effect of classmates’ social support in mitigating adverse disease outcome is congruent with reports by Von Weiss et al. [45] and Varni et al. [46] among children with rheumatic disease and newly diagnosed cancer, respectively. This beneficial effect of classmates’ social support is understandable considering that a majority of the index study population were adolescents. Consistent with a developmental point of view, various facets of adolescent life are more influenced by peer factors/opinions than family factors [47, 48]. Furthermore, classmates (peers) provide empathy and emotional support that enables adaptive coping skills and cushion the disease trajectory. The implication is that there is need to explore evidence-based methods probably through structured education and modelling to improve positive relationships within the class or peers.

The positive effect of higher parental support on SCA severity demonstrated in this study has been reported elsewhere [22]. Though children with chronic disorders remain dependent on their families for care and support, their over protectiveness often leads to restrictions and loss of autonomy which the child desires [49]. Despite this, the manner in which the child perceives parental support can help them cope with their disease [49]. The former could thus be responsible for the negative correlation between SCA severity scores, and the parental support subscale scores obtained in this study.

In the teacher’s domain, there was a weak relationship with SCA severity. Studies have shown that there is a high variability in support offered to children with sickle cell disease by teachers [50, 51]. Teachers have been reported to lack empathy and knowledge of sickle cell disease [50]. In addition, adolescents commonly do not disclose their condition to their teachers due to fear of alienation and stigmatization [49, 52]. This often led to a lack of support from those teachers whose input could otherwise have helped them get through crisis and stigmatization. In summary, there is some consensus that social support, on the whole, is beneficial from many different perspectives as shown in the index study.

Regarding perceived stress scores, the index study found a weak relationship with SCA severity. A significant relationship between stress and some indices of SCA severity has been reported by several authors [13, 17, 18, 20, 53, 54]. Porter et al. [53, 54] reported a temporal relationship between stress and painful crisis. Similarly, Gil et al. [17] noted that higher levels of stress was associated with increased same day pain. This relationship between stress and sickle cell pain was corroborated by Shah et al. [18] in 2020. There is evidence that psychological stress causes parasympathetic withdrawal and sympathetic activation and a glucocorticoid hormone response, both of which culminate in reduced microvascular blood flow and pro-inflammatory response leading to vaso-occlusive events [18, 21]. However, painful crisis which is just an item in the global scoring system used in the index study was the sole marker of disease severity used in the above studies and this limits the generalizability of the findings. Moreso, the very low co-efficient of determination obtained in this study shows that only 0.3% of the variation in sickle cell severity was contributed by a change in perceived stress. This was also observed by Thompson [13] that perceived stress acting alone may not be enough of an independent mediator of disease severity in SCA but may act additively with other factors. This addictive effect is such that the cumulative effect of perceived stress and other mediators is greater than the sum of their individual effect. The relationship between perceived stress and disease severity may also have been weakened by the absence in this study of participants with severe disease. Furthermore, the use of parent proxy report of perceived stress for younger participants may have influenced the scores in this study. Perceived stress is a subjective feeling and as such best reported by the individual, also parental mood has been shown to influence proxy reports [55].

### Limitations

First, the use of clinic-based samples (care seekers); although it saved cost and time, and is the predominant methodology in the literature, a community sample may be more representative. Second, the confounding effects of the different medications such as disease modifying agents (e.g., hydroxyurea) were not controlled for.

## Conclusion

The findings of this study support the available literature on the possible positive relationship between satisfactory perceived social support and lower SCA severity. These findings buttress the need for clinicians to consider psychosocial methods (e.g., family and classmates’ support) in improving disease outcomes.

## Electronic supplementary material

Below is the link to the electronic supplementary material.

Supplementary Material 1

## Data Availability

The data was attached as a supplementary file.

## Abbreviations

CASSS: The Child and Adolescent Social Support Scale
HPLC: High Performance Liquid Chromatography
HbF: Foetal Haemoglobin
IBM: SPSS–International Business Machine–Statistical Package for Social Sciences
n: Final sample size
p: Estimated proportion of an attribute that is present in the population
q: 1–p
e: Desired level of Precision
z: Abscissa of the Normal Curve (i.e., 95% Confidence Interval)
PSS: The Perceived Stress Scale
SCA: Sickle Cell Anaemia
UNTH: University of Nigeria Teaching Hospital

## References

[1] Abhulimhen-Iyoha BI, Israel-Aina YT, Joel-Utomakili K. Sickle cell anaemia: morbidity profile and outcome in a paediatric emergency setting in Nigeria. Afri J Med Health Sci. 2015;14(2):79–82.

[2] Olatunya OS, Ogundare EO, Fadare JO, Oluwayemi IO, Agaja OT, Adeyefa BS, et al. The financial burden of sickle cell disease on households in Ekiti, Southwest Nigeria. Clinicoecon Outcomes Res. 2015;7(21):545–53. 26622186 10.2147/CEOR.S86599 PMC4639479

[3] Singh R, Jordan R, Hanlon C. Economic impact of sickle cell hospitalization. Blood. 2014;124(21):5971.

[4] Piel FB, Patil AP, Howes RE, Nyangiri OA, Gething PW, Dewi M, et al. Global epidemiology of sickle haemoglobin in neonates: a contemporary Geostatistical model-based map and population estimates. Lancet. 2013;381(9861):142–51. 23103089 10.1016/S0140-6736(12)61229-X PMC3547249

[5] World Health Organization. Fifty ninth World Health Assembly. Sickle cell anemia: Report by the Secretariat. Provisional agenda 2006, Item 11.4. Available at www.apps.who.int/iris/handle/10665/20890, accessed on 3rd November, 2019.

[6] Van den Tweel XW, Van Der Lee JH, Heijboer H, Peters M, Fijnvandraat K. Development and validation of a pediatric severity index for sickle cell patients. Am J Hematol. 2010;85(10):746–51. 20806231 10.1002/ajh.21846

[7] Makani J, Ofori-Acquca SF, Nnodu O, Wonkem A, Onene-Fiempong K. Sickle cell disease: new opportunities and challenges in Africa. Sci World J. 2013. 10.1155/2013/193252. 10.1155/2013/193252 PMC3988892 25143960

[8] Quinn CT. Mini-review: clinical severity in sickle cell disease: the challenges of definition and prognostication. Exp Biol Med. 2016;241(7):679–88. 10.1177/1535370216640385 PMC4871738 27013545

[9] Adegoke SA, Kuti BP. Evaluation of clinical severity of sickle cell anemia in Nigerian children. J Appl Hematol. 2013;4(2):58–64.

[10] Alabid T, Kordofani AA, Atalla B, Bebekir A, Elamin BK. Evaluation of clinical severity of sickle cell anaemia in Sudanese patients. Am J Res Commun. 2016;4(6):23–5.

[11] Tewari S, Brousse V, Piel FB, Menzel S, Rees DC. Environmental determinants of severity in sickle cell disease. Haematologica. 2015;100(9):1108–16. 26341524 10.3324/haematol.2014.120030 PMC4800688

[12] Engel GL. The clinical application of the biopsychosocial model. J Med Philos. 1981;6(2):101–24. 7264472 10.1093/jmp/6.2.101

[13] Thompson (Jr) RJ. The interaction of social, behavioral, and genetic factors in sickle cell disease. In Genes, Behavior, and the Social Environment: Moving Beyond the Nature/Nurture Debate. National Academies Press (US). 2006. Available at https://www.ncbi.nlm.nih.gov/books/NBK19938/, accessed on the 5th of September, 2021. 20669442

[14] Havelka M, Despot Lučanin J, Lučanin D. Biopsychosocial model–the integrated approach to health and disease. Coll Antropol. 2009;33(1):303–10. 19408642

[15] Vaughn LM, McLinder D, Jacquez F, Crosby L, Slater S, Mitchell M. Understanding the social networks of parents of children with sickle cell disease. J Health Care Poor Underserved. 2011;22(3):1014-29. 10.1353/hpu.2011.0087 PMC4877700 21841293

[16] Burlew K, Telfair J, Colangelo L, Wright EC. Factors that influence adolescent adaptation to sickle cell disease. J Pediatr Psycho. 2000;25(5):287–99. 10.1093/jpepsy/25.5.287 10880059

[17] Gil KM, Carson JW, Porter LS, Ready J, Valrie C, Redding-Lallinger R, et al. Daily stress and mood and their association with pain, health-care use, and school activity in adolescents with sickle cell disease. J Pediatr Psychol. 2003;28(5):363–73. 12808013 10.1093/jpepsy/jsg026

[18] Shah P, Khaleel M, Thuptimdang W, Sunwoo J, Veluswamy S, Chalacheva, et al. Mental stress causes vasoconstriction in subjects with sickle cell disease and in normal controls. Haematologica. 2020;105(1):83–90. 10.3324/haematol.2018.211391. 30975906 10.3324/haematol.2018.211391 PMC6939522

[19] Barakat LP, Lash LA, Lutz MJ, Nicolaou DC. Psychosocial adaptation of children and adolescents with sickle cell disease. Comprehensive handbook of childhood cancer and sickle cell disease: A biopsychosocial approach. United Kingdom: Oxford Press Scholarship Online; 2020. pp. 471–95. 10.1093/oso/9780195169850.001.0001.

[20] Valrie CR, Gil KM, Redding-Lallinger R, Daeschner C. Brief report: daily mood as a mediator or moderator of the pain–sleep relationship in children with sickle cell disease. J Pediatr Psychol. 2007;33(3):317–22. 17690117 10.1093/jpepsy/jsm058

[21] Xu C, Lee SK, Zhang D, Frenette PS. The gut Microbiome regulates psychological-stress-induced inflammation. Immunity. 2020;53(2):417–28. 32735844 10.1016/j.immuni.2020.06.025 PMC7461158

[22] Sehlo MG, Kamfar HZ. Depression and quality of life in children with sickle cell disease: the effect of social support. BMC Psychiatry. 2015;15(1):78. 10.1186/s12888-015-0461-6. 25880537 10.1186/s12888-015-0461-6 PMC4394397

[23] Tempier R, Balbuena L, Lepnurm M, Craig TK. Perceived emotional support in remission: results from an 18-month follow-up of patients with early episode psychosis. Soc Psychiatry Psychiatr Epidemiol. 2013;48(12):1897–904. 23661149 10.1007/s00127-013-0701-3

[24] Akinsheye I, Alsultan A, Solovieff N, Ngo D, Baldwin CT, Sebastiani P, et al. Fetal hemoglobin in sickle cell anemia. Blood. 2011;118(1):19–27. 21490337 10.1182/blood-2011-03-325258 PMC3139383

[25] Adeodu OO, Akinlosotu MA, Adegoke SA, Oseni SB. Foetalhaemoglobin and disease severity in Nigerian children with sickle cell anaemia. Mediterr J Hematol Infect Dis. 2017;9(1):23–6. 10.4084/MJHID.2017.063 PMC5667525 29181140

[26] Fatunde OJ, Scott-Emuakpor AB. Foetalhaemoglobin in Nigerian children with sickle cell anaemia. Effect on haematological parameters and clinical severity. Trop Geogr Med. 1992;44(3):264–46. 1280870

[27] Olaniyi JA, Arinola OG, Odetunde AB. Foetalhaemoglobin (HbF) status in adult sickle cell anaemia patients in Ibadan, Nigeria. Ann Ib Postgrad Med. 2010;8(1):30–3. 25161472 10.4314/aipm.v8i1.63955 PMC4138770

[28] Aygun B, Odame I. A global perspective on the sickle-cell disease. Pediatr Blood Cancer. 2012;59(2):386–90. 10.1002/pbc.24175. 22535620 10.1002/pbc.24175

[29] Diwe K, Iwu AC, Uwakwu K. Prevalence and pattern of sickle cell disease among children attending tertiary and non-tertiary healthcare institutions in a southeastern state. JRMDS. 2016;4:31–3.

[30] Cochran WG. Sampling Techniques, 2nd edition, New York: John Wiley and Sons, Inc. 1963.

[31] Oyedeji GA. Socio-economic and cultural background of hospitalized children in ilesa. Niger J Paediatr. 1985;12:111–7.

[32] Cohen S, Kamarck T, Mermelstein R. Perceived stress scale. Measuring stress: A guide for health and social scientists. In: Sanad HM. Stress and anxiety among junior nursing students during the initial clinical training: a descriptive study at College of Health Sciences, University of Bahrain. Am. J. Nurs Res. 2019;6:995–9.

[33] Cohen S. Perceived stress in a probability sample of the united States. In: Spacapan S, Oskamp S, editors. The social psychology of health. California: Sage Publications, Inc.; 1988. pp. 31–67.

[34] Liu X, Zhao Y, Li J, Dai J, Wang X, Wang S. Factor structure of the 10-Item perceived stress scale and measurement invariance across genders among Chinese adolescents. Front Psychol. 2020;11:537. 10.3389/fpsyg.2020.00537. 32328009 10.3389/fpsyg.2020.00537 PMC7160845

[35] Perera MJ, Brintz CE, Birnbaum-Weitzman O, Penedo FJ, Gallo LC, Gonzalez P, et al. Factor structure of the perceived stress Scale-10 (PSS) across english and Spanish Language responders in the HCHS/SOL Sociocultural ancillary study. Psychol Assess. 2017;29(3):320–8. 27280744 10.1037/pas0000336 PMC5148735

[36] Kupst MJ, Butt Z, Stoney CM, Griffith JW, Salsman JM, Folkman S, et al. Assessment of stress and self-efficacy for the NIH toolbox for neurological and behavioral function. Anxiety Stress Coping. 2015;28(5):531–44. 25577948 10.1080/10615806.2014.994204 PMC4515370

[37] Ameringer S, ElswickJr RK, Smith W. Fatigue in adolescents and young adults with sickle cell disease: biological and behavioral correlates and health-related quality of life. J Pediatr Oncol Nurs. 2014;31(1):6–17. 24378816 10.1177/1043454213514632 PMC3982311

[38] Ossai EN, Alo AT, Onwe BC, Okoro DO, Ezeagu NE, Ogbonnaya LU. Prevalence and predictors of perceived stress: A study among medical students of Ebonyi state university Abakaliki, Nigeria. Asian J Adv Basic Sci. 2019;30:1–9.

[39] Oku AO, Owoaje ET, Oku OO, Ikpeme BM. Prevalence of stress, stressors and coping strategies among medical students in a Nigerian medical school. Afr J Med Health Sci. 2015;14(1):29.

[40] Asani MO, Farouk Z, Gambo S. Prevalence of perceived stress among clinical students of Bayero University Medical School. NJBCS. 2016;13(1):55–62.

[41] Malecki CK, Demaray MK. Measuring perceived social support: development of the child and adolescent social support scale (CASSS). Psychol Sch. 2002;39(1):1–8.

[42] College of American Pathologist. Haemoglobinopathy. In Laboratory Accreditation Program: Haematology and Coagulation Checklist. 2019;131–135.

[43] IBM Corp. Released 2011. IBM SPSS statistics for windows, version 20.0. Armonk, NY: IBM Corp.

[44] Imhonde HO, Ndom RJ. Social-support, self-esteem and depression as determinants of quality of life among sickle cell patients. IFE Psychologia. 2013;21(1):101–13.

[45] von Weiss RT, Rapoff MA, Varni JW, Lindsley CB, Olson NY, Madson KK, et al. Daily hassles and social support as predictors of adjustment in children with pediatric rheumatic disease. J Pediatr Psychol. 2002;27(2):155–65. 11821499 10.1093/jpepsy/27.2.155

[46] Varni JW, Katz ER, Colegrove R, Dolgin M. Perceived social support and adjustment of children with newly diagnosed cancer. J Dev Behav Pediatr. 1994;15(1):20–6. 8195433 10.1097/00004703-199402000-00004

[47] Tome G, de Malos MG, Simoes C, Camacho I, AlvesDiniz J. How can peer group influence the behavior of adolescents: explanatory model? Global J Health Sci. 2012;4(2):26–35. 10.5539/gjhs.v4n2p26 PMC4777050 22980148

[48] Albert D, Chein J, Steinberg L. Peer influences on adolescent decision making. Curr Dir Psychol Sci. 2013;22(2):114–20. 25544805 10.1177/0963721412471347 PMC4276317

[49] Ola B, Olajide A, Olajide S, Williamson IR, Dyson SM. An education session on sickle cell disease with teachers in Lagos, Nigeria: evaluation of a randomized controlled trial. Int J Educ Res. 2018;5(3):115–24.

[50] Lightfoot J, Mukherjee S, Sloper P. Supporting pupils with special health needs in mainstream schools: policy and practice. Child Soc. 2001;15(2):57–69.

[51] Dyson SM, Atkin K, Culley LA, Dyson SE, Evans H, Rowley DT. Disclosure and sickle cell disorder: a mixed methods study of the young person with sickle cell at school. Soc Sci Med. 2010;70(12):2036–44. 20385437 10.1016/j.socscimed.2010.03.010

[52] Moraes B, Ngomah-Moraes A, Munsaka E. Lived experiences of adolescent learners with sickle cell disease. Health Press Zambia Bull. 2019;3(5):4–11.

[53] Porter LS, Gil KM, Sedway JA, Ready J, Workman E, Thompson RJ. Pain and stress in sickle cell disease: an analysis of daily pain records. Int J Behav Med. 1998;5(3):185–203. 16250701 10.1207/s15327558ijbm0503_1

[54] Porter LS, Gil KM, Carson JW, Anthony KK, Ready J. The role of stress and mood in sickle cell disease pain: an analysis of daily diary data. J Health Psychol. 2000;5(1):53–63. 22048823 10.1177/135910530000500109

[55] Abate C, Lippé S, Bertout L, Drouin S, Krajinovic M, Rondeau É, et al. Could we use parent report as a valid proxy of child report on anxiety, depression, and distress? A systematic investigation of father–mother–child triads in children successfully treated for leukemia. Pediatr Blood Cancer. 2018;65(2):26840. 10.1002/pbc.26840 29049860

